# Prevalence and Risk Factors for Post-Traumatic Stress Reaction Among Resident Survivors of the Tsunami That Followed the Great East Japan Earthquake, March 11, 2011

**DOI:** 10.1017/dmp.2016.18

**Published:** 2016-04-14

**Authors:** Chieko Matsubara, Hitoshi Murakami, Koubun Imai, Tetsuya Mizoue, Hidechika Akashi, Chiaki Miyoshi, Tamotsu Nakasa

**Affiliations:** 1 Bureau of International Medical Cooperation, National Center for Global Health and Medicine, Tokyo, Japan; 2 Department of Psychiatry, National Center for Global Health and Medicine, Tokyo, Japan; 3 Department of Epidemiology and Prevention, National Center for Global Health and Medicine, Tokyo, Japan.

**Keywords:** tsunami, post-traumatic stress reaction, Great East Japan Earthquake, resident survivor

## Abstract

**Objective:**

The Great East Japan Earthquake triggered a massive tsunami that devastated the coasts of northern Japan on March 11, 2011. Despite the large number of “resident survivors,” who have continued to reside on the upper floors of damaged houses, few studies have examined the mental health of these residents. We explored the prevalence and risk factors of post-traumatic stress reaction (PTSR) among resident survivors.

**Methods:**

A cross-sectional household screening for health support needs was conducted among resident survivors in Higashi-Matsushima city, Miyagi, 2 to 4 months after the tsunami. Questions assessing PTSR were included in the screening interviews.

**Results:**

Of 5103 resident survivors, 5.7% experienced PTSR. PTSR risk factors, identified via regression analysis, differed according to the height of house flooding. When house flooding remained below the ground floor, PTSR was significantly associated with being female and regular psychotropic medication intake. These 2 factors in addition to being middle-aged or elderly and living alone were also risk factors when flood levels were above the ground floor.

**Conclusions:**

Following the tsunami, PTSR was found in a considerable number of resident survivors. Attention and support for people who use psychiatric medication, their families, and people living alone are suggested as possible directions for public health strategies. (*Disaster Med Public Health Preparedness*. 2016;page 1 of 8)

On the afternoon of March 11, 2011, the Great East Japan Earthquake (a magnitude 9.0 undersea earthquake with an epicenter around 70 km off Japan’s Pacific Coast) triggered a massive tsunami that caused widespread devastation in the coastal areas of northern Japan. The tsunami’s waves reached heights of 40 meters. By March 11, 2013, 15,882 people had been confirmed dead, with a further 2668 missing.^1^ Drowning was the predominant cause of death (90.6%).^2^

In the coastal city of Higashi-Matsushima, Miyagi Prefecture, 65% of urban areas were engulfed by the tsunami, which traveled as far as 3 km inland. Inundation rates of Higashi-Matsushima city were the highest of the municipalities hit by the tsunami.^3^ On September 1, 2014, the city office reported that 1109 of its 43,000 residents had died. In total, 97% of the houses were completely or almost completely destroyed; 76% and 21% were completely and almost completely destroyed, respectively.^3^

In the wake of the tsunami, as many as 1 in 3 of the city’s residents were displaced, and evacuation centers were seriously overcrowded.^4^ Some survivors, however, remained in their damaged homes, while others only returned to their houses from the evacuation centers once the floodwaters had subsided. Where lower levels had been destroyed by the tsunami, these “resident survivors” were often forced to live on the upper floors of their homes.

There were few resident survivors in the inundation areas, because the tsunami destroyed approximately 75% of the houses in the locations in which its height reached 0.5 m and more than 80% of the houses in locations in which its height reached beyond 2.5 m.^5^ Moreover, distribution channels and medical institutions in the city were severely damaged and lifeline utilities cut off. Anecdotal reports indicated that resident survivors struggled with insufficient medical care and emergency support in their homes, many of which were almost entirely covered in mud at the ground level.^4^

In areas inundated by the tsunami, with the exception of regions in which all homes had been destroyed, the local health office conducted household screening for health support needs.^4^ The health interview conducted with resident survivors evaluated their mental and physical health status with the explicit priority of identifying all health service needs. Screened survivors were then referred to regional medical facilities, where the necessary health services were provided. Such services included examination by medical doctors, examination by psychiatric specialists, prescription of medication, and home-care assistance.

Post-traumatic stress disorder (PTSD) is a significant concern among those directly affected by disasters. Being female is commonly known to be one of the predictors of PTSD following disasters such as earthquakes,^6^ ^,^ ^7^ tsunamis,^8^ and tornados.^9^ Other predictors of PTSD have been found to be loss of livelihood,^5^ ^,^ ^8^ witnessing others’ suffering,^10^ direct exposure to the disaster,^8^ ^,^ ^10^ ^,^ ^11^ older age,^6^ ^,^ ^8^ ^,^ ^12^ and loss of possessions.^7^ ^,^ ^13^

Studies into the aftermath of tsunamis that followed the massive undersea earthquakes in Indonesia in 2004 and on the northern coast of Japan in 2011 reported greater prevalence of PTSD among displaced persons than observed in nondisplaced persons.^14^ ^-^ ^16^ However, PTSD was shown to decline over time.^8^ ^,^ ^14^ ^,^ ^17^

Previous studies have also reported that strong social ties are linked to a lower risk of the development of mental health problems.^18^ ^-^ ^22^ After trauma, social support is inversely related to PTSD.^13^ ^,^ ^21^ ^,^ ^23^ To date, receiving social support has been shown to have negatively affected the development of PTSD in tourists at 6 months after the tsunami;^8^ however, little is known regarding resident survivors during subacute periods following a tsunami or how social ties affect the mental health of such individuals when vital community links are severely devastated.

In order to design and implement more effective public health response measures, there is a need to better understand the impact of tsunamis on the mental health of resident survivors. To address this issue, this study aimed to explore the prevalence and risk factors of post-traumatic stress reaction (PTSR) among resident survivors of the tsunami caused by the Great East Japan Earthquake of 2011.

## Methods

We conducted a cross-sectional household screening for health support needs.

### Study Sites

Thirty-one completely or partially flooded local administrative areas were targeted for household screening. Overall, there were 7084 registered households in the study area, 4672 (59.9%) of which were subsequently confirmed to be inhabited via the screening.^4^

### Data Collection

Household screening began on April 26, 2011, and was completed by July 29, 2011. Screening was conducted by public health nurses from Higashi-Matsushima city and other regions offering assistance to local health officers. The screening was rolled out from areas with the most severe destruction, because demand in those areas was deemed the greatest.

### The Target Population

The target population of this study comprised individuals who continued to reside in their damaged houses in targeted tsunami-flooded areas, who were aged 15 years or older, and who were contactable, in person, at home. In some cases, individuals whose houses were not flooded were also included in the screening, because of the manner in which the census was administered in some regions of the city. The exclusion criteria were having stayed at evacuation centers or having moved outside the flooded areas, being aged less than 15 years, and not being able to be contacted at home. Screening involving those aged less than 18 years was only conducted once parental consent was obtained.

In total, 5455 residents aged 15 years and older were contacted, in person, at home; however, a total of 15,503 registered residents of all ages were screened. Primarily because of work or school commitments, more than half of the household members were not present at the time the surveys were conducted. By extension, those who were included in the daytime screenings were most likely to have been those who were home during the tsunami.

### Questionnaire

A questionnaire was administered to participants and gathered demographic data such as age and sex, as well as other personal information such as physical status (eg, any chronic medical conditions and medication), mental status (including depressive reaction and PTSR), and the direct impact of the tsunami on their homes (including flooding in the house; disruption of lifeline utilities such as water, electricity, and gas; and direct exposure to seawater). During previous visits, public health nurses had determined that survivors were reluctant to talk about lost employment or deceased or missing family members; therefore, these issues were not included in the questionnaire.

### Dependent Variables

The dependent variable in this study was PTSR, which was assessed by using the core variables from the *Diagnostic and Statistical Manual of Mental Disorders, Fourth Edition* (DSM-IV)^24^ selected by psychiatric specialists. The selection rationale was supported by a higher lifetime prevalence of symptoms of criteria B, C, and D associated with trauma.^25^ A 3-item measure was applied for this screening. These core variables were included in the household screening, mainly because of tight time constraints and the wide range of responses that were to be obtained. Psychiatric specialists did not include criteria A1 or E in the DSM-IV, because all of the survivors were exposed to an extreme traumatic stressor involving direct personal experience of an event that threatened death (ie, the tsunami, which was criterion A1), and the duration of the disturbance appeared to exceed 1 month (criterion E). Streamlining the questions also allowed the psychiatrists to identify those individuals who needed immediate diagnosis and treatment more quickly.

The following 3 items were used to screen individuals for PTSR:

1) Do you react with inappropriate sensitivity to trivial noises or movement?
2) Do you avoid places, people, and topics that remind you of the earthquake and/or the tsunami?
3) Do you think about the tsunami even though you would rather forget it?

Respondents who agreed with all items 1, 2, and 3 were deemed to be suffering from PTSR.

### Independent Variables

Age and sex were collected as demographic data in this study. Age was categorized into 3 groups (15-39 years, 40-64 years, and 65 years and older) in accordance with the criteria outlined by the World Health Organization for defining people of middle age and the elderly.^26^

The number of cohabitants was measured as an indicator of social ties in this study. Cohabiting families, communities, schools, and places of work often provide social ties, but the tsunami destroyed many community links, and resident survivors were sporadically distributed across devastated areas. Not wishing to distress the respondents, the public health nurses decided not to ask questions related to previous community, school, or workplace ties. Cohabitants were categorized into tertile groups in order to examine whether this variable was significantly associated with the risk of onset of PTSR.

Height of house flooding and direct exposure to seawater were recorded as indicators of the impact of the tsunami on resident survivors. The height of house flooding was categorized into 3 groups in descending order of severity: above the ground floor, below the ground floor, and no flooding. The mortality rates were classified into 5 categories according to place of residence. The availability of lifeline utilities (water, electricity, and gas) under post-disaster conditions was also assessed and adjusted to examine the impact of the tsunami on PTSR, because the availability of these services was expected to affect the behavior and attitudes of the respondents.

### Analysis

Data were analyzed by using SPSS statistics version 21 for Windows (IBM Corp, Armonk, NY). Chi-square tests for univariate analyses were conducted. Stepwise logistic regression was performed to explore the risk factors of PTSR, using all variables simultaneously stratified according to height of house flooding. Regression analyses were adjusted by the number of weeks that had elapsed between the tsunami and the screening and by mortality rate at the place of residence.^27^ Statistical significance was set at *P*<0.05.

### Ethical Considerations

The Research Ethics Committee of the National Center for Global Health and Medicine, Japan, reviewed and approved all screening procedures (ethical approval code: NCGM-G-00110300). The National Center for Global Health and Medicine acted in collaboration with the city of Higashi-Matsushima with regard to the restoration of public health. Participants were informed about the screening objectives and procedure via verbal explanation, and written consent was obtained before enrolment. Eighty-seven household members declined to participate in the screening.

## Results

The results of the univariate analysis of PTSR in the respondents are shown in Table 1. More respondents were female (63.0%) and aged 65 years or older (36.9%) relative to those in the city population (51.0% and 22.6%, respectively). A total of 81.1% of the respondents lived in flooded houses in totally or partially flooded administrative areas. Approximately 10% of the respondents lived alone. The prevalence of PTSR was 5.7%.

**Table 1:** Univariate Analysis of Post-Traumatic Stress Response Among Resident Survivors of the Tsunami That Followed the Great East Japan Earthquake, March 11, 2011

	**Post-Traumatic Stress Response**		
	**No. of Respondents** ^**a**^	**%**	***P*** **-Value** ^**b**^
**Total**	5454	5.7	
**Mortality rate at place of residence**			
0.00% to 0.50%	1765	6.4	0.237
0.51% to 1.00%	1,934	5.8	
1.01% to 5.0%	1257	4.7	
5.01% to 10.00%	0	0	
≥10.01%	127	4.7	
**House flooding**			
Above the ground floor	3187	6.4	0.021
Below the ground floor	631	6.0	
No flooding	888	3.9	
**Sex**			
Male	1666	6.4	0.002
Female	3437	4.3	
**Age**			
15 to 39 years old	889	3.5	0.006
40 to 64 years old	2201	6.4	
65 years old and over	2013	5.9	
**Direct exposure to seawater**			
Yes	573	7.7	0.027
No	4433	5.4	
**Regular intake of psychotropic medicine(s)**			
Yes	172	14.0	<0.001
No	4931	5.4	
**Number of cohabitants**			
More than 6 persons	279	4.3	0.125
1 to 6 person(s)	4331	5.6	
None	493	7.5	
**Water supply**			
Yes	4539	5.8	0.945
No	175	5.7	
**Electricity supply**			
Yes	4578	5.8	0.937
No	107	5.6	
**Gas supply**			
Yes	4330	5.8	0.620
No	261	6.5	

a Number may not add up to 5454 because some of the respondents did not answer all of the questions.

b Chi-square test.

The results of the univariate analysis of PTSR, stratified according to the height of house flooding, are shown in Table 2. PTSR was not significantly associated with variables for respondents whose houses were not flooded. Female resident survivors living in houses flooded both below (*P* = 0.019) and above (*P* = 0.031) the ground floor were more likely to experience PTSR. Regular intake of psychotropic medications (*P*<0.001), direct exposure to seawater (*P* = 0.018), and age (*P* = 0.011) also predicted PTSR among people whose houses were flooded above the ground floor.

**Table 2:** Univariate Analysis of Post-Traumatic Stress Reaction for House Flooding Strata Among Resident Survivors of the Tsunami That Followed the Great East Japan Earthquake on March 11, 2011 (n=4706)

	**Post-Traumatic Stress Reaction**														
	**No Flooding** ^**a**^ **(n=888)**					**Below the Ground Floor** ^**a**^ **(n=631)**					**Above the Ground Floor** ^**a**^ **(n= 187)**				
	**Yes**		**No**			**Yes**		**No**			**Yes**		**No**		
	**No.**	**%**	**No.**	***%***	***P*** **-value** ^**b**^	**No.**	**%**	**No.**	***%***	***P*** **-value** ^**b**^	**No.**	**%**	**No.**	***%***	***P*** **-value** ^**b**^
**Sex**															
Male	9	3.0	296	97.0	0.273	6	2.9	203	97.1	0.019	55	5.1	1020	94.9	0.031
Female	26	4.5	557	95.5		32	7.6	390	92.4		150	7.1	1962	92.9	
**Age**															
15 to 39 years	4	2.2	176	97.8	0.274	5	5.5	86	94.5	0.564	19	3.6	508	96.4	0.011
40 to 64 years	16	5.1	296	94.9		13	5.0	247	95.0		107	7.3	1353	92.7	
≥65 years	15	3.8	381	96.2		20	7.1	260	92.9		79	6.6	1121	93.4	
**Direct exposure to seawater**															
Yes	0	0	30	100	0.627	0	0	45	100	0.101	42	9.1	422	90.9	0.018
No	35	4.1	812	95.9		38	6.6	540	93.4		159	5.9	2515	94.1	
**Regular intake of psychotropic medicine(s)**															
Yes	3	11.1	24	88.9	0.086	3	18.8	13	81.3	0.065	16	14.4	95	85.6	<0.001
No	32	3.7	829	96.3		35	5.7	580	94.3		189	6.1	2887	93.9	
**Number of cohabitants**															
More than 6 persons	3	5.4	53	94.6	0.770	1	2.9	33	97.1	0.482	8	5.4	140	94.6	0.059
1 to 6 person(s)	28	3.7	720	96.3		35	6.5	504	93.5		166	6.1	2545	93.9	
None	4	4.8	80	95.2		2	3.4	56	96.6		31	9.5	297	90.5	
**Water supply**															
Yes	31	3.8	782	96.2	0.289	36	5.9	572	94.1	0.299	197	6.5	2851	93.5	0.521
No	4	6.8	55	93.2		2	11.1	16	88.9		4	4.3	90	95.7	
**Electricity supply**															
Yes	35	4.1	823	95.9	0.635	35	5.8	571	94.2	0.555	194	6.4	2850	93.6	0.463
No	0	0	11	100		0	0	10	100		6	7.1	79	92.9	
**Gas supply**															
Yes	35	4.1	814	95.9	0.535	36	6.1	558	93.9	0.397	178	6.3	2644	93.7	0.274
No	0	0	15	100		0	0	15	100		17	7.5	209	92.5	

a Number may not be add up to 888, 631, or 3187 because not all the respondents answered all the questions.

b Chi-square test or Fisher exact test.

The results of the stepwise logistic regression analysis of factors associated with PTSR, stratified according to degree of house flooding, are shown in Table 3. None of these factors significantly predicted PTSR for the respondents whose houses were not flooded. For the resident survivors whose houses were flooded below the ground floor, the odds of having PTSR were 3.8 times (95% confidence interval [CI]: 1.01-14.01) and 2.5 times (95% CI: 1.01-6.11) greater for those with a regular intake of psychotropic medication and for females, respectively. For the resident survivors whose houses were flooded above the ground floor, the odds of having PTSR were 2.5 times greater among respondents who regularly used psychotropic medications (95% CI: 1.39-4.30), approximately 2 times greater for middle-aged respondents (95% CI: 1.16-3.26), and 1.6 times greater in respondents directly exposed to seawater (95% CI: 1.10-2.31) and in female respondents (95% CI: 1.11-2.20). Conversely, the odds of having PTSR were 2.9 and 1.8 times lower in respondents living with 1 to 6 (adjusted odds ratio [AOR] = 0.35; 95% CI: 0.13-0.94) and more than six (AOR = 0.57; 95% CI: 0.37-0.88) other people, respectively. The R^2^ was 0.04 for houses flooded both below and above the ground floor.

**Table 3:** Final Model of the Stepwise Logistic Regression Analysis of Factors Associated With Post-Traumatic Stress Reaction^a^

		**Post-Traumatic Stress Reaction (n=4634)**				
		B	SE	*P*-value	AOR	95% CI
**Below the ground floor**						
Gender						
	Male	Ref.				
	Female	0.91	0.46	0.047	2.49	(1.01-6.11)
Regular intake of psychotropic medication						
	No	Ref.				
	Yes	1.32	0.67	0.049	3.76	(1.01-14.01)
**Above the ground floor**						
						
Week		−0.11	0.03	<0.001	0.90	(0.85-0.95)
Gender						
	Male	Ref.				
	Female	0.45	0.17	0.010	1.56	(1.11-2.20)
Age						
	15 to 39 years	Ref.				
	40 to 64 years	0.67	0.26	0.011	1.95	(1.16-3.26)
	≥65 years	0.45	0.27	0.096	1.58	(0.92-2.69)
Direct exposure to sea water						
	No	Ref.				
	Yes	0.47	0.19	0.014	1.59	(1.10-2.31)
Regular intake of psychotropic medication						
	No	Ref.				
	Yes	0.90	0.29	0.002	2.45	(1.39-4.30)
Number of cohabitants						
	None	Ref.				
	1 to 6 person(s)	−0.56	0.22	0.011	0.57	(0.37-0.88)
	More than 6 persons	−1.05	0.50	0.037	0.35	(0.13-0.94)

a Abbreviations: SE, standard error; AOR, adjusted odds ratio; CI, confidence interval.

## Discussion

The results of this study demonstrated a PTSR prevalence of 5.7% in the resident survivors at 2 to 4 months (the subacute period) after the March 11 tsunami. To our knowledge, this is the first study to examine PTSR in resident survivors following a tsunami. The DSM-IV diagnostic criteria were partly applied in this study; however, the results revealed that the prevalence of PTSR was much higher in respondents than it was in the general population, as shown in the benchmark World Mental Health Japan Survey 2002-2003, which also used the DSM-IV criteria (the prevalence rate of PTSD within the preceding year was 0.4%; 95% CI: 0.0-0.8).^28^

Vulnerability to PTSR in the resident survivors was associated with the height of house flooding. Tsunami height changes significantly according to slight changes in coastal topography. For example, the height of a tsunami increases sharply when the tsunami reaches shallow coasts or narrow bays; therefore, some inner areas were hit by the tsunami independent of their distance from the coast, because the tsunami traveled along the rivers.^29^ Therefore, the height of house flooding could be considered one of the predictors of PTSR in resident survivors of a tsunami; this is unlike earthquakes and terrorist attacks for which distance from the epicenter^30^ and site of the attack,^13^ respectively, predict PTSD.

In addition, the height and speed of the flood affect residents’ ability to evacuate because it is more difficult to walk through fast-running water. During a huge typhoon in Japan in 1959, the height of the flooding made it difficult for evacuees to walk. Male adults, female adults, and children aged 10 to 12 years experienced difficulty in walking through the water at depths of approximately 0.7 m, 0.5 m, and 0.2 m, respectively.^31^ The estimated rate of death caused by a tsunami increases sharply when the depth of the floodwater exceeds 0.7 m (0.01% at 0.3 m, 4.8% at 0.5 m, 71.1% at 0.7 m, and 100% at 1.0 m), based on past records of tsunamis.^5^ Furthermore, the height of the ground floor is generally approximately 0.5 m in Japanese houses;^32^ therefore, house flooding of a height over 0.5 m (eg, 0.7 m) was close to marginal with respect to evacuation. For these reasons, many of the resident survivors who lived in houses flooded above the ground floor might have been exposed to a high risk of peritraumatic distress, which occurs at the time of and immediately following trauma. Peritraumatic distress is known to be one of the predictors of PTSD; ^23^ ^,^ ^33^ therefore, it would be preferable if future studies on the present topic also use a peritraumatic distress scale.^34^

The results of this study showed that being female was significantly associated with PTSR, which was consistent with the results of previous studies.^6^ ^-^ ^9^ Given the lack of stability that women relative to men experience when walking through running water, which is influenced by the strength and height of the water flow,^35^ peritraumatic distress caused by the perception of difficulty in achieving safe evacuation may cause more severe PTSR in women than in men.

The determinants of PTSR experienced by resident survivors differed according to the height of house flooding. As flood height increased, the number of factors predicting PTSR also increased in this study. For example, resident survivors who used psychotropic medication regularly and lived in houses flooded below and above the ground floor were 3.8 and 2.5 times more likely to experience PTSR, respectively. However, this significant association was not found between resident survivors who used psychotropic medication regularly but did not experience house flooding. For middle-aged resident survivors who lived in houses flooded above the ground floor and experienced direct exposure to seawater, the number of weeks that had elapsed since the occurrence of the tsunami was significantly associated with experiencing PTSR. There are 2 possible reasons for this.

First, anecdotal reports indicated that although evacuees wished to remain in the evacuation centers, they found it difficult to do so, owing to health problems, such as autism, alcoholism, and schizophrenia, experienced personally or by their family members. For the resident survivors whose houses were flooded above the ground floor, returning to their homes at the time of the flood or immediately thereafter would have caused a considerable degree of peritraumatic distress.^23^ Results also indicated that for the resident survivors whose houses were not flooded, regular intake of psychotropic medication was not associated with PTSR. Therefore, there is a clear need for special support for resident survivors who regularly used psychotropic medication, particularly those whose houses are flooded above the ground floor, as this would be helpful in reducing the incidence of PTSR in this group.

Second, on the basis of the stratified regression analysis of PTSR, factors that have been shown to be predictors of PTSD in numerous previous studies, such as gender, were not observed in respondents whose houses were not flooded. One possible explanation was voiced by health personnel who worked in inundated areas: the resident survivors appeared to confront their lives and environments in a beneficial manner and possessed a greater ability to cope with stressors. These remarks may represent a sense of coherence, a concept developed by the health sociologist Antonovsky.^36^ A former study in Japan showed that possessing a sense of coherence was positively associated with good mental health.^37^ Future studies should examine sense of coherence in resident survivors by use of a validated scale.^38^ PTSD risk could be managed more effectively if an approach is found to reduce respondents’ burden during data collection by using variables such as employment loss or the death of family members.

The results of this study demonstrated that cohabitation could alleviate PTSR in resident survivors whose houses were flooded above the ground floor; resident survivors were 2 and 3 times less likely to experience PTSR if they lived with 1 to 6 and more than 6 people, respectively. Similarly, weaker social ties predicted PTSD following a terrorist attack^13^ and aircraft accident.^39^ In communities in which many houses had been damaged or destroyed, resident survivors did not receive sufficient social support and were prone to isolation. Although people differ according to their family circumstances, and some people prefer to live alone, the findings indicate that social support should be provided to resident survivors who live alone in devastated areas, regardless of their backgrounds.

The association between the number of weeks since the occurrence of the tsunami and PTSR decreased according to the time that had elapsed since the tsunami. These results were consistent with previous studies^14^ ^,^ ^17^ ^,^ ^40^ and suggested that PTSR would diminish over time.

We noted some limitations, which may have had an effect on the scope and generalizability of the results. Although identifying a number of variables from the DSM-IV criteria allowed us to focus in depth on these factors in our study, it also presented a limitation with respect to the extent to which we were able to learn about respondent survivors’ mental health. In future studies, the full use of validated scales, with fewer questions, that can be applied in household screening should be considered. In this study, the people who met the definition of PTSR were referred to visiting medical and care systems for further assessment and treatment. Second, a greater proportion of elderly women were included in this study than in the city’s population. These elderly female residents were also more likely to have been affected by the tsunami while at home, as the earthquake happened at 2:46 PM. Even though we had reasons for the choice of design in the present study, a random sampling survey might have been more representative of the population by including all households without limited penetration (eg, of people working and out of the house). Third, the screening was conducted earlier in areas in which destruction was greater; therefore, the regression analysis may have been strongly influenced by the number of weeks that had elapsed since the tsunami. Fourth, the questionnaire did not include any variables concerning deceased or missing family members; lost jobs; depth of exposure to the tsunami; variables related to the survivors’ social networks, other than number of cohabitants; or variables related to emotional responses experienced during and immediately after the tsunami, such as peritraumatic distress,^32^ because it was apparent in the pre-interviews that the respondents were reluctant to answer such questions. Future studies that include these variables are required if an approach can be found to make such inquiries less burdensome. Fifth, this study could not reveal causality, because of the nature of cross-sectional studies.

## Conclusions

The results suggest that PTSR is found in a considerable number of resident survivors following a tsunami. The predictors of PTSR differed according to the height of house flooding. PTSR was significantly associated with female sex and regular intake of psychotropic medication in resident survivors whose houses were flooded below the ground floor. These 2 factors, in addition to fewer weeks having elapsed since the tsunami, being middle-aged or elderly, direct exposure to seawater, and living alone, were also associated with experiencing PTSR in resident survivors whose houses were flooded above the ground floor. Facing the aftermath of an unprecedented tsunami, considerable effort was made to increase resilience, despite the limited health resources; however, the results of this study suggest that resident survivors still had a high risk of developing PTSR. Further attention and support for people living with psychiatric medication, their families, and people living alone are suggested as a possible direction for public health strategies following a devastating tsunami so as to prevent the development of PTSR in resident survivors and maximize the impact of the limited available health resources.

## References

[1] The National Police Agency. Police measures and damage situation of the Tohoku-Pacific Ocean Earthquake, 2011 (as of 11 March, 2013) (in Japanese). http://www.npa.go.jp/archive/keibi/biki/higaijokyo.pdf. Published 2014. Accessed September 28, 2014.

[2] Cabinet Office, Government of Japan. White paper on disaster management 2011 (in Japanese). http://www.bousai.go.jp/kaigirep/hakusho/h23/bousai2011/html/honbun/index.htm. Published 2011. Accessed September 28, 2014.

[3] Higashi-Matsushima city. The situation of the earthquake damage (as of 1 September, 2014) (in Japanese). http://www.city.higashimatsushima.miyagi.jp/cnt/saigai_20110311/index.html. Published 2014. Accessed September 28, 2014.

[4] Department of Health and Welfare, Higashi-Matsushima city, Miyagi Prefecture. Overcome together the Great East Japan Earthquake: Report of public health nurse, dietitian activity at Higashi-Matsushima city (in Japanese). Published 2013.

[5] Cabinet Office, Government of Japan. Estimation of the tsunami heights, the inundation areas and damage after the Nankai Trough Great Earthquake (in Japanese). http://www.bousai.go.jp/jishin/nankai/nankaitrough_info.html. Published 2012. Accessed September 28, 2014.

[6] Fan F, Zhang Y, Yang Y, et al Symptoms of posttraumatic stress disorder, depression, and anxiety among adolescents following the 2008 Wenchuan Earthquake in China. J Trauma Stress. 2011;24(1):44-53. doi: 10.1002/jts.20599;10.1002/jts.20599. 21351164

[7] Zhang Z, Ran MS, Li YH, et al. Prevalence of post-traumatic stress disorder among adolescents after the Wenchuan Earthquake in China. *Psychol Med*. 2012;42(8):1687-1693. doi: 10.1017/S0033291711002844. 22152150

[8] Hussain A, Weisaeth L, Heir T. Posttraumatic stress and symptom improvement in Norwegian tourists exposed to the 2004 tsunami--a longitudinal study. BMC Psychiatry. 2013;13:232-244X-13-232. doi: 10.1186/1471-244X-13-232. 24063414 PMC3851444

[9] Adams ZW, Sumner JA, Danielson CK, et al Prevalence and predictors of PTSD and depression among adolescent victims of the spring 2011 tornado outbreak. J Child Psychol Psychiatry. 2014;55(9):1047-1055. doi: 10.1111/jcpp.12220. 24580551 PMC4133357

[10] Rosendal S, Salcioglu E, Andersen HS, et al Exposure characteristics and peri-trauma emotional reactions during the 2004 tsunami in Southeast Asia--what predicts posttraumatic stress and depressive symptoms? Compr Psychiatry. 2011;52(6):630-637. doi: 10.1016/j.comppsych.2010.12.004;10.1016/j.comppsych.2010.12.004. 21349509

[11] Kristensen P, Weisaeth L, Heir T. Psychiatric disorders among disaster bereaved: an interview study of individuals directly or not directly exposed to the 2004 tsunami. Depress Anxiety. 2009;26(12):1127-1133. doi: 10.1002/da.20625;10.1002/da.20625. 19998267

[12] Chen H, Chen Y, Au M, et al The presence of post-traumatic stress disorder symptoms in earthquake survivors one month after a mudslide in southwest china. *Nurs Health Sci*. 2014;16(1):39-45. doi: 10.1111/nhs.12127. 24635896

[13] Galea S, Ahern J, Resnick H, et al Psychological sequelae of the September 11 terrorist attacks in New York City. N Engl J Med. 2002;346(13):982-987. doi: 10.1056/NEJMsa013404. 11919308

[14] van Griensven F, Chakkraband ML, Thienkrua W, et al Mental health problems among adults in tsunami-affected areas in southern Thailand. JAMA. 2006;296(5):537-548. doi: 10.1001/jama.296.5.537. 16882960

[15] Udomratn P. Prevalence of tsunami-related PTSD and MDD in Thailand. Asian J Psychiatr. 2009;2(4):124-127. doi: 10.1016/j.ajp.2009.10.008;10.1016/j.ajp.2009.10.008. 23051090

[16] Tayama J, Ichikawa T, Eguchi K, et al Tsunami damage and its impact on mental health. *Psychosomatics*. 2012;53(2):196-197. doi: 10.1016/j.psym.2011.11.005;10.1016/j.psym.2011.11.005. 22424169

[17] Thavichachart N, Tangwongchai S, Worakul P, et al Posttraumatic mental health establishment of the tsunami survivors in Thailand. Clin Pract Epidemiol Ment Health. 2009;5:11-0179-5-11. doi: 10.1186/1745-0179-5-11;10.1186/1745-0179-5-11. 19490651 PMC2702267

[18] Kawachi I, Berkman LF. Social ties and mental health. *J Urban Health*. 2001;78(3):458-467. doi: 10.1093/jurban/78.3.458. 11564849 PMC3455910

[19] Schweitzer R, Melville F, Steel Z, et al Trauma, post-migration living difficulties, and social support as predictors of psychological adjustment in resettled Sudanese refugees. *Aust N Z J Psychiatry*. 2006;40(2):179-187. doi: 10.1111/j.1440-1614.2006.01766.x. 16476137

[20] Cluver L, Fincham DS, Seedat S. Posttraumatic stress in AIDS-orphaned children exposed to high levels of trauma: the protective role of perceived social support. *J Trauma Stress*. 2009;22(2):106-112. doi: 10.1002/jts.20396;10.1002/jts.20396. 19319917

[21] Seplaki CL, Goldman N, Weinstein M, et al Before and after the 1999 Chi-Chi earthquake: traumatic events and depressive symptoms in an older population. Soc Sci Med. 2006;62(12):3121-3132. doi: 10.1016/j.socscimed.2005.11.059. 16423437

[22] Harville EW, Xiong X, Smith BW, et al Combined effects of Hurricane Katrina and Hurricane Gustav on the mental health of mothers of small children. J Psychiatr Ment Health Nurs. 2011;18(4):288-296. doi: 10.1111/j.1365-2850.2010.01658.x. 21418428 PMC3472438

[23] Ozer EJ, Best SR, Lipsey TL, et al Predictors of posttraumatic stress disorder and symptoms in adults: a meta-analysis. *Psychol Bull*. 2003;129(1):52-73. 12555794 10.1037/0033-2909.129.1.52

[24] The American Psychiatric Association. Diagnostic and Statistical Manual of Mental Disorders, 4th ed Washington, DC: American Psychiatric Association; 1994.

[25] Breslau N, Peterson EL, Kessler RC, et al Short screening scale for DSM-IV posttraumatic stress disorder. *Am J Psychiatry*. 1999;156(6):908-911. 10360131 10.1176/ajp.156.6.908

[26] World Health Organization. Definition of an older or elderly person. http://www.who.int/healthinfo/survey/ageingdefnolder/en/. Accessed September 28, 2014.

[27] Tani K. Distribution of the number of deaths and the death rate on the Great East Japan Earthquake. Occasional paper of Department of Geography, Saitama University. 2012(32):1-26.

[28] Kawakami N, Takeshima T, Ono Y, et al Twelve-month prevalence, severity, and treatment of common mental disorders in communities in Japan: Preliminary finding from the World Mental Health Japan survey 2002-2003. Psychiatry Clin Neurosci. 2005;59(4):441-452. doi: 10.1111/j.1440-1819.2005.01397.x. 16048450

[29] Miyatake K. Countermeasures of river management facilities for earthquake and tsunami based on the Great East Japan Earthquake (in Japanese). JICE Report: Report of Japan Institute of Construction Engineering. 2011 (20):39-46.

[30] Basoglu M, Kilic C, Salcioglu E, et al Prevalence of posttraumatic stress disorder and comorbid depression in earthquake survivors in Turkey: an epidemiological study. *J Trauma Stress*. 2004;17(2):133-141. doi: 10.1023/B:JOTS.0000022619.31615.e8. 15141786

[31] Ministry of Land, Infrastructure, Transport and Tourism. Guidelines for disaster management of inundation in underground space (in Japanese). http://www.mlit.go.jp/river/basic_info/jigyo_keikaku/saigai/tisiki/chika/. Accessed September 28, 2014.

[32] National Institute for Land and Infrastructure Management. River glossary (in Japanese). http://www.nilim.go.jp/lab/rcg/newhp/yougo/words/043/043.html. Accessed September 28, 2014.

[33] Nishi D, Koido Y, Nakaya N, et al Peritraumatic distress, watching television, and posttraumatic stress symptoms among rescue workers after the Great East Japan Earthquake. *PLoS One*. 2012;7(4):e35248. doi: 10.1371/journal.pone.0035248;10.1371/journal.pone.0035248. 22558130 PMC3338412

[34] Brunet A, Weiss DS, Metzler TJ, et al The peritraumatic distress inventory: a proposed measure of PTSD criterion A2. *Am J Psychiatry*. 2001;158(9):1480-1485. 11532735 10.1176/appi.ajp.158.9.1480

[35] Suga K, Uesaka T, Yoshida T, et al Preliminary study of feasible safe evacuation in flood disaster. Annual Journal of Hydraulic Engineering. 1995;39:879-882.

[36] Antonovsky A. Unraveling the Mystery of Health: How People Manage Stress and Stay Well. Jossey-Bass; 1987.

[37] Tsuno YS, Yamazaki Y. Relationships among sense of coherence, resources, and mental health in urban and rural residents in Japan. *BMC Public Health*. 2012;12:1107-2458-12-1107. 23259917 10.1186/1471-2458-12-1107 PMC3552666

[38] Togari T, Yamazaki Y, Nakayama K, et al Development of a short version of the sense of coherence scale for population survey. *J Epidemiol Community Health*. 2007;61(10):921-922. doi: 61/10/921. 17873231 10.1136/jech.2006.056697 PMC2652976

[39] Fullerton CS, Ursano RJ, Kao TC, et al Disaster-related bereavement: acute symptoms and subsequent depression. *Aviat Space Environ Med*. 1999;70(9):902-909. 10503757

[40] Frankenberg E, Friedman J, Gillespie T, et al Mental health in Sumatra after the tsunami. *Am J Public Health*. 2008;98(9):1671-1677. doi: 10.2105/AJPH.2007.120915;10.2105/AJPH.2007.120915. 18633091 PMC2509591

